# THC for age-related cognitive decline?

**DOI:** 10.18632/aging.101648

**Published:** 2018-11-12

**Authors:** Yosef Sarne

**Affiliations:** 1 Department of Physiology and Pharmacology, Sackler Faculty of Medicine, Tel Aviv University, Tel Aviv, Israel

**Keywords:** tetrahydrocannabinol, cognitive impairment, Sirtuin 1, drug of abuse, medical marijuana

Cognitive decline is a painful aspect of normal aging, with enormous clinical, social and economic implications. No effective treatment is available for this aggravating condition, even though preventive measures such as physical exercise, mental activity and balanced diet have been suggested to slow down the cognitive deterioration that begins already in midlife. Last year, Bilkei-Gorzo and co-workers showed that a chronic treatment with tetrahydrocannabinol (THC; the main psychoactive ingredient of cannabis) restored cognitive functions in aging mice [1]. This study was in accordance with previous findings on chronic treatment with the synthetic cannabinoid agonist WIN-55212-2 that improved memory in old rats [2]. In these two studies, the experimental rodents were infused with either 3 mg/kg of THC or 2 mg/kg of WIN-55212-2 per day for either 28 or 21 days, respectively.

Cannabis (marijuana) is still defined as an illicit drug of abuse and previous reviews have described the deleterious outcomes of chronic cannabis use (see, for example [3]). On the other hand, a growing number of countries and states recognize cannabis and its active ingredients (the phytocannabinoids) as beneficial for the treatment of various clinical disorders (see [4] for a recent review). Synthetic THC ("dronabinol") is already approved for the treatment of nausea and vomiting in people undergoing chemotherapy and is available as oral capsules ("Marinol") in doses of 2.5-10 mg. The oromucosal spray nabiximols ("Sativex") that is authorized in several countries for treating multiple sclerosis spasticity, contains 2.7 mg of THC per spray. "Medical marijuana" is approved for various clinical indications in doses of 1-20 mg that correspond to the range of self-titrated doses smoked by users of recreational marijuana. Considerable rates of adverse effects have been reported following the continuous use of cannabis for medical purposes [5]. Even a single exposure to a conventional dose of THC can impair memory, orientation, attention and motivation for several hours. This interference with optimal functioning in everyday life represents a substantial disadvantage especially to the elderly fragile patient.

Recently, we found that a single administration of an extremely low dose of THC to old mice improved their cognitive performance in a battery of six different behavioral assays that measured different aspects of memory and learning [6]. The old mice were injected with 0.002 mg/kg of THC, a dose that was 1000 times lower than the conventional doses of THC (1-10 mg/kg) that induced the conventional cannabinoid effects in rodents. THC-treated old mice performed significantly better than vehicle-treated old mice, and had a similar performance to young control mice. The beneficial effects of the single injection of THC lasted for at least 7 weeks and was accompanied by long-lasting biochemical and structural changes in the brain. Thus, the level of Sirtuin 1 was elevated in the hippocampus and frontal cortex for several weeks following treatment with THC. Sirtuin 1 is a NAD-dependent protein deacetylase that was previously shown to be involved in synaptic plasticity, memory formation and neuronal development, and to mediate the neuroprotective effects of resveratrol, melatonin and calorie restriction. Furthermore, magnetic resonance imaging (MRI) revealed that the single administration to mice of ultra-low THC increased the volume of the entorhinal cortex, the prefrontal cortex and the posterior hippocampus, and increased tissue density in 11 regions of the old brain [6]. Further experiments in our laboratory showed that this ultra-low dose of THC induced neurogenesis in the hippocampus and olfactory bulb of the aging mice (Sarne et al, in preparation).

Can these surprising neuroprotective effects of ultra-low THC in mice be translated to clinical treatment in aging humans? An appropriate conversion of drug doses from experimental animals to humans should be normalized for body surface area [7]. Hence, the dose of 0.002 mg/kg in mice is equivalent to 0.0002 mg/kg in humans, yielding a dose of 0.014 mg (or 14 micrograms) for an average patient of 70 kg body weight. This extremely low dose is 100 times lower than the threshold for the conventional mental and somatic effects of THC. The exact range of therapeutic doses, as well as the required frequency of administration, awaits clinical investigation.
